# Amyloid precursor like protein-1 promotes JNK-mediated cell migration in *Drosophila*

**DOI:** 10.18632/oncotarget.17681

**Published:** 2017-05-08

**Authors:** Xingjun Wang, Ying Sun, Shilong Han, Chenxi Wu, Yeqing Ma, Yu Zhao, Yingyao Shao, Yujun Chen, Lingzhi Kong, Wenzhe Li, Fan Zhang, Lei Xue

**Affiliations:** ^1^ Department of Interventional Radiology, Shanghai 10th People's Hospital, Shanghai Key Laboratory of Signaling and Diseases Research, School of Life Science and Technology, Tongji University, Shanghai 200092, China; ^2^ College of Chinese Medicine, North China University of Science and Technology, Tangshan 063210, China; ^3^ Clinical Translational Research Center, Shanghai Pulmonary Hospital, School of Life Science and Technology, Tongji University, Shanghai 200092, China

**Keywords:** APLP1, cell migration, *Drosophila*, JNK

## Abstract

The amyloid precursor like protein-1 (APLP1) is a member of the amyloid precursor protein (APP) family in mammals. While many studies have been focused on the pathologic role of APP in Alzheimer's disease, the physiological functions of APLP1 have remained largely elusive. Here we report that ectopic expression of APLP1 in *Drosophila* induces cell migration, which is suppressed by the loss of JNK signaling and enhanced by the gain of JNK signaling. APLP1 activates JNK signaling through phosphorylation of JNK, which up-regulates the expression of matrix metalloproteinase MMP1 required for basement membranes degradation and promotes actin remodeling essential for cell migration. Our data thus provide the first *in vivo* evidence for a cell-autonomous role of APLP1 protein in migration.

## INTRODUCTION

Amyloid precursor-like protein 1 (APLP1) is a member of the highly conserved amyloid precursor protein family that includes amyloid precursor protein (APP) and amyloid precursor-like protein 2 (APLP2) in mammals [1–4]. Members of this family are type I integral membrane proteins that contain a single transmembrane domain with a large N-terminal extracellular domain and a short C-terminal intracellular domain (AICD) [5]. While APP has been studied extensively as the precursor of amyloid beta (Aβ) peptides implicated in the pathogenesis of Alzheimer's disease (AD) [6], the physiological functions of APP have yet to be fully elucidated. Previous studies have suggested a role of APP in neurogenesis [7, 8], neurite outgrowth [9–12], axonal pruning [13], synapse formation and function [14–16], cargo transport along the axon [17–20], cell death [21] and cell fate determination [22].

APP family proteins have also been implicated in neuronal migration, yet this function has been controversial. It was originally reported that neurons in APP/APLP1/APLP2 triple knockout mice over-migrated and accumulated in the marginal zone [23], suggesting that APP family proteins negatively regulate neuronal migration. Yet, APP triple knockout neurons differentiated from APP triple knockout embryonic stem (ES) cells have unaltered migratory capacities both *in vivo* and *in vitro* [24], and down-regulation of APLP2 in APP/APLP1 double knock mice did not affect the migration of cortical neurons [25]. RNAi knock-down of APP in the developing cortex blocked the migration of neurons from the intermediate zone to the cortical plate [26], suggesting a positive role of APP in neuronal migration. However, this phenotype was not resulted from a general defect in cell motility, rather, a specific defect in the interaction between APP ectodomain and the extracellular factors, such as pancortins were detected [27]. Furthermore, cultured CHO cells overexpressing APLP2 exhibited an enhanced migratory response to fibronnectin and type IV collagen through increased adhesion to these extracellular molecules [28]. Together, these studies suggest that APP and APLP2 are required for cell adhesion with extracellular factors, yet a direct role of APP family proteins in promoting cell migration has not been documented.

Though APP family proteins have conserved structure and could compensate for each other to certain extent in double and triple knockout mice [23], APLP1 likely carries out distinct physiological functions from APP and APLP2. First, APLP1 is mainly localized to the cell surface, whereas APP and APLP2 are mostly found in intracellular compartments [29]. Second, APLP1, but not APP and APLP2, interacts in trans and is involved in cell-cell contacts [29]. Third, APLP1 does not undergo the same type of regulated processing as APP and APLP2 [30]. Despite a few studies on the processing and expression of APLP1, the functions of APLP1 remain largely unknown. APLP1 is reported to be a p53 transcriptional target that modulates stress-induced apoptosis [31], and is up-regulated in neuroendocrine tumors of the gastrointestinal tract [32].

The c-Jun N-terminal Kinase (JNK) pathway is evolutionarily conserved from *Drosophila* to human, and regulates a wide range of cellular activities including proliferation, differentiation, migration and apoptosis [33]. Recently, JNK pathway was shown to play important roles in modulating Src-induced cell migration [34] and Aβ induced cell death in *Drosophila* [35, 36]. However, a functional connection between JNK and APLP1 has not been established.

*Drosophila* has been used as an animal model to study the *in vivo* function of APP, for the genetics of *Drosophila* is clear and *Drosophila* is easy to manipulate. In *Drosophila*, there is also an APP-like protein, APPL, indicating the role of APPL may be conserved from *Drosophila* to human. In this report, we investigate the function of APLP1 in *Drosophila*. We found that expression of APLP1 in *Drosophila* wing disc triggers cell migration, which is suppressed by the loss of JNK signaling and enhanced by the gain of JNK signaling. APLP1 activates JNK signaling through phosphorylation of JNK, which results in elevated expression of JNK target genes including *puc* and *mmp1*, and actin polymerization. Thus, this work provides the first evidence that APLP1 promotes JNK-dependent cell migration *in vivo*.

## RESULTS

### APLP1 induces cell migration in *Drosophila* wing discs

**Figure 1:**
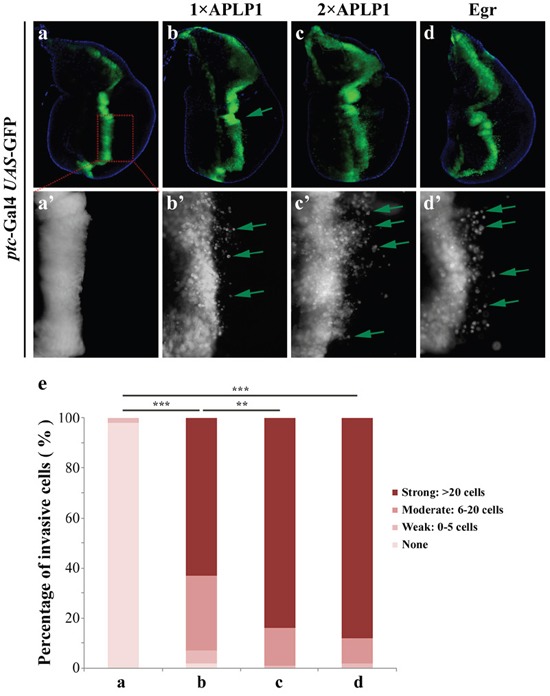
APLP1 induces cell migration in *Drosophila* Fluorescence micrographs of wing discs are shown. Compared with the *ptc*-Gal4 *UAS*-GFP control (**a**, **a'**), expression of APLP1 induced moderate cell migration behavior (**b**, **b'**) that was enhanced by adding another copy of APLP1 **(c**, **c')**. As a positive control, expression of Egr produced a similar cell migration phenotype (**d**, **d'**). **a'**-**d'** are high magnifications of **a**-**d**. (**e**) Quantification of migration phenotype. The migration phenotype was classified into four groups based on the number of GFP-labeled cells migrated to the posterior compartment. None: no GFP-labeled cells; Weak: 1-5 cells; Moderate: 6-20 cells; Strong: >20 cells. More than 20 discs were examined for each genotype. The crosses were performed at 29°C.***, P<0.001; **, P<0.01. The genotypes used in the figure are as follows: *ptc*-Gal4 *UAS*-GFP/+ (**a**, **a'**), *ptc*-Gal4 *UAS*-GFP/+; *UAS*-APLP1/+ (**b**, **b'**), *ptc*-Gal4 *UAS*-GFP/+; *UAS*-APLP1/*UAS*-APLP1 (**c**, **c'**) and *ptc*-Gal4 *UAS*-GFP/+; *UAS*-Egr/+ (**d**, **d'**).

To investigate the physiological functions of APLP1 in development, we adopted the Gal4/UAS system to express APLP1 in various tissues in *Drosophila*. Interestingly, expression of APLP1 along the anterior/posterior (A/P) compartment boundary in third-instar wing discs under the control of *patched*-Gal4 (Figures 1a and 1a'), a driver commonly used to study cell migration behavior in *Drosophila* [37, 38], produced cell migration phenotype. The green fluorescent protein (GFP) labeled cells are detached from the A/P compartment boundary and migrates toward the posterior part of the wing discs (Figures 1b and '). This phenotype was further enhanced by adding another copy of *UAS*-APLP1 (Figures 1c and 1c'), as the number of discs showing strong migration was increased from 63% to 84% (Figure 1e).

The JNK signaling was reported to play a pivotal role in regulating cell migration in *Drosophila* [33, 39, 40]. Consistently, activation of JNK signaling by expressing the *Drosophila* TNF ortholog Egr [41, 42], or the JNK kinase Hep [43], produces a similar migration phenotype (Figures 1d, 1d' and 1e and data not shown). Thus, expression of APLP1 in *Drosophila* recapitulates that of JNK activation and promotes cell migration.

### JNK signaling is required for APLP1-induced cell migration

**Figure 2:**
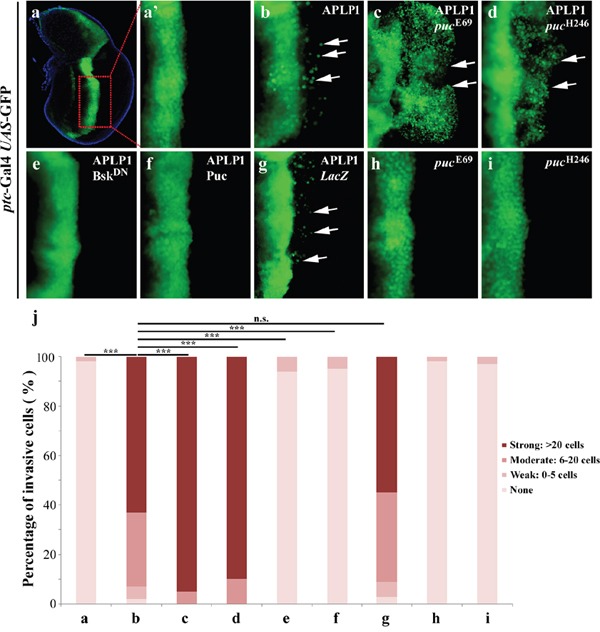
APLP1-induced cell migration depends on JNK signaling Fluorescence micrographs of wing discs are shown. Compared with the *ptc*-Gal4 *UAS*-GFP control (**a**, **a'**), APLP1-induced cell migration (**b**) was enhanced in heterozygous *puc* mutants (**c**, **d**), and was blocked by expression of Bsk^DN^ (**e**) or Puc (**f**), but not by that of *LacZ* (**g**). The *puc* mutants did not produce migration phenotype by themselves (**h**, **i**). (**j**) Quantification of migration phenotype in **a**-**i**. The crosses were performed at 29°C. ***, P <0.001, n.s., no significance. The genotypes used in the figure are as follows: *ptc*-Gal4 *UAS*-GFP/+ (**a**, **a'**), *ptc*-Gal4 *UAS*-GFP/+; *UAS*-APLP1/+ (**b**), *ptc*-Gal4 *UAS*-GFP/+; *UAS*-APLP1/*puc*^E69^ (**c**), *ptc*-Gal4 *UAS*-GFP/+; *UAS*-APLP1/*puc*^H246^ (**d**), *ptc*-Gal4 *UAS*-GFP/+; *UAS*-APLP1/*UAS*-Bsk^DN^ (**e**), *ptc*-Gal4 *UAS*-GFP/+; *UAS*-APLP1/*UAS*-Puc (**f**), *ptc*-Gal4 *UAS*-GFP/+; *UAS*-APLP1/*UAS*-*LacZ* (**g**), *ptc*-Gal4 *UAS*-GFP/+; *puc*^E69^/+ (**h**) and *ptc*-Gal4 *UAS*-GFP/+; *puc*^H246^/+ (**i**).

To investigate whether JNK signaling is involved in APLP1-induced cell migration, we first enhanced the JNK signaling by mutating one cope of endogenous *puckered* (*puc*) gene. *puc* encodes a JNK phosphatase that negatively regulates JNK activity [44, 45]. We found that APLP1-induced cell migration phenotype (Figure 2b) was significantly exacerbated by two independent *puc* mutants (Figures 2c and 2d), which by themselves produced no evident cell migration phenotype (Figures 2h and 2i). As a positive control, *ptc*> Egr induced cell migration was also enhanced by *puc* mutant (Supplementary Figure 1d). Thus, gain of JNK signaling could exaggerate APLP1-induced cell migration.

To examine whether loss of JNK signaling could suppress APLP1-triggered cell migration, we expressed a dominant negative form of Bsk (Bsk^DN^) encoding the *Drosophila* JNK [46], or the JNK phosphatase Puc. APLP1-induced cell migration was completely blocked by expression of Bsk^DN^ (Figures 2e and 2j) or Puc (Figures 2f and 2j), but not by that of *LacZ* (Figures 2g and 2j). Consistently, the *puc* mutant-enhanced APLP1-induced cell migration phenotype was also suppressed by expression of Bsk^DN^ (Supplementary Figures 1e and 1g) or Puc (Supplementary Figures 1f and 1g). In conclusion, JNK signaling is absolutely required for APLP1-induced cell migration in *Drosophila*.

We also checked APLP2, amyloid precursor-like protein 2, in cell migration. Expression of APLP2 induced moderate cell migration phenotype (data not shown), which was consistent with APLP1 in cell migration, indicating a universal role of APLPs in cell migration. To examine whether APLP1 induced JNK-mediated cell migration is conserved from *Drosophila* to human beings, we checked APPL, a *Drosophila* APP-like protein [47], in cell migration. Consistent with our hypothesis, expression of APPL driven by *ptc*-Gal4 induced significant cell migration phenotype, which was suppressed by expression of Bsk^DN^ (Supplementary Figures 2c, 2c' and 2f) or Puc (Supplementary Figures 2d, 2d' and 2f) and exacerbated by *puc* mutant (Supplementary Figures 2e, 2e' and 2f). Collectively, the results revealed that APPL and APLP1 induced JNK-mediated cell migration, indicating cell migration function of APLP1 is conserved and is not obtained later in evolution.

### APLP1 induces JNK phosphorylation and *puc* activation in *Drosophila*

**Figure 3:**
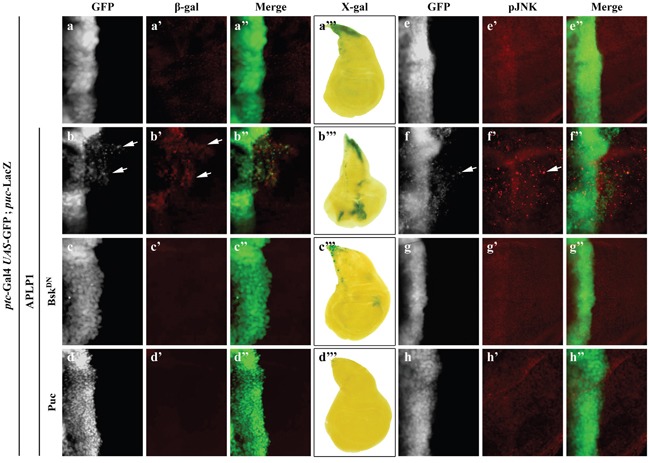
APLP1 activates JNK signaling *in vivo* Fluorescence (**a-a”**, **b-b”**, **c-c”**, **d-d”**, **e-e”**, **f-f”**, **g-g”**, **h-h”**) and light (**a'''**, **b'''**, **c'''**, **d'''**) micrographs of wing disc are shown. Compared with the *ptc*-Gal4 *UAS*-GFP control (**a**-**a'''**, **e-e”**), expression of APLP1 activated *puc* expression, detected by β-gal antibody (**b-b”**) or X-gal staining (**b'''**), and JNK phosphorylation (**f**-**f''**), which were suppressed by expression of Bsk^DN^ (**c**-**c'''**, **g**-**g''**) or Puc (**d**-**d'''**, **h**-**h''**). The crosses were performed at 25°C. The genotypes used in the figure are as follows: *ptc*-Gal4 *UAS*-GFP/+; *puc*-LacZ/+ (**a-a”'**, **e-e''**), *ptc*-Gal4 *UAS*-GFP/+; *puc*-LacZ/*UAS*-APLP1 (**b-b”'**, **f-f''**), *ptc*-Gal4 *UAS*-GFP/+; *puc*-LacZ/*UAS*-APLP1 *UAS*-Bsk^DN^ (**c-c'''**, **g-g''**), *ptc*-Gal4 *UAS*-GFP/+; *puc*-LacZ/*UAS*-APLP1 *UAS*-Puc (**d-d'''**, **h-h''**).

The above data indicates that APLP1 is able to trigger JNK-dependent cell migration in *Drosophila*. To check if APLP1 could activate JNK signaling *in vivo*, we examined the expression of *puc*-LacZ reporter, a read-out of JNK signaling [45, 48], and JNK phosphorylation in the wing discs. Compared with the control (Figures 3a-3a''', 3e-3e''), expression of APLP1 induced both *puc-*LacZ expression (Figures 3b-3b''') and JNK phosphorylation (Figures 3f-3f''), which were blocked by expression of Bsk^DN^ (Figures 3c-3c''', 3g-3g'') or Puc (Figures 3d-3d''', 3h-3h''), suggesting APLP1 is able to activate JNK signaling *in vivo*.

To investigate whether APLP1 could activate JNK signaling in different cellular contexts, we directed APLP1 expression by other Gal4 drivers. We found that APLP1 expression driven by *engrailed*-Gal4 (*en*-Gal4) triggered *puc*-LacZ expression (Supplementary Figures 3c-3c''') and JNK phosphorylation (Supplementary Figures 3d-3d'') in the posterior compartment of wing discs, which was suppressed by the expression of Bsk^DN^ (Supplementary Figures 3e-3e'', 3f-3f''). In addition, APLP1 expression in the wing pouch driven by *scalloped*-Gal4 (s*d*-Gal4) or in the salivary gland also activates JNK signaling, resembling that of Hep expression (Supplementary Figure 4). Collectively, the data indicate that APLP1 is able to trigger JNK activation in various cellular contexts in *Drosophila*. Consistently, expression of *Drosophila* APPL also upregulated JNK signaling in the wing discs (Supplementary Figure 5b-5b'''), demonstrating APLP1 induced JNK activation is conserved in evolution.

### APLP1 induces JNK mediated MMP1 expression and actin polymerization

**Figure 4:**
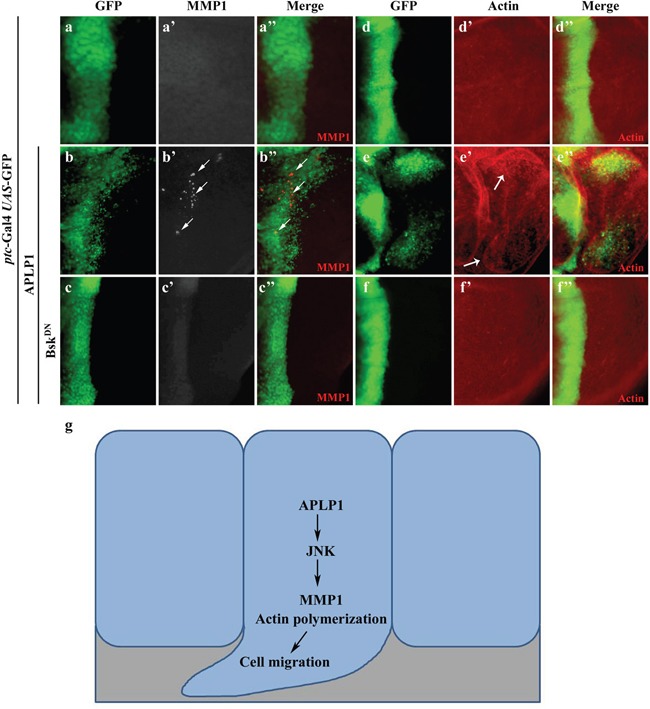
APLP1 induces JNK-dependent MMP1 expression and actin remodeling Fluorescence micrographs of wing discs are shown (**a-f**). Compared with the *ptc*-Gal4 *UAS*-GFP control (**a-a''**, **d-d”**), expression of APLP1 activated MMP1 expression (**b-b''**) and actin polymerization (**e-e”**, indicated by white arrows), both were blocked by expression of Bsk^DN^ (**c-c''**, **f**-**f''**). A model for APLP1-induced JNK-dependent cell migration was shown (**g**). The crosses were performed at 29°C. The genotypes used in the figure are as follows: *ptc*-Gal4 *UAS*-GFP/+; *puc*-LacZ/+ (**a-a''**, **d**-**d”**), *ptc*-Gal4 *UAS*-GFP/+; *puc*-LacZ/*UAS*-APLP1 (**b-b''**, **e**-**e”**), *ptc*-Gal4 *UAS*-GFP/+; *puc*-LacZ/*UAS*-APLP1 *UAS*-Bsk^DN^ (**c-c''**, **f**-**f''**).

Previous studies have suggested that JNK-dependent cell migration is mediated by transcriptional up-regulation of the matrix metalloproteinase MMP1, which is required for the degradation of basement membranes [33, 49–53]. Consistently, expression of APLP1 driven by *ptc*-Gal4 up-regulated MMP1 expression in the A/P boundary and posterior compartment (Figures 4a-4a'', 4b-4b''), which was blocked by inactivation of JNK signaling (Figures 4c-4c''). Intriguingly, APLP1 expression driven by *en*-Gal4 triggered GFP-labeled cells to migrate from the posterior compartment toward anteriorly with elevated MMP1 expression (Supplementary Figures 6a-6a'', 6b-6b''), a phenotype that was inhibited by inactivation of JNK signaling (Supplementary Figures 6c-c''). Previous work suggested JNK signaling-dependent actin remodeling is required for cell migration [53, 54]. Consistent with its ability to activate JNK signaling, APLP1 expression induced actin polymerization in *ptc* domain and migrated cells (Figures 4d-4d”, 4e-4e''), which was abrogated by blocking JNK signaling (Figures 4f-4f''). Taken together, APLP1 induces JNK-dependent MMP1 expression and actin polymerization, which are required for basement membranes degradation and cell motility, respectively [52, 55, 56]. Consistently, downregulation of MMP1 and MMP2 or expression of tissue inhibitor of matrix metalloprotease-1 (TIMP1) [57–59] compromised APLP1 induced cell migration phenotype (Supplementary Figures 7b-7f). Rho GTPases play an important role in diverse biological processes such as actin cytoskeleton organization [60, 61]. As expected, loss of Rho-1 moderately suppressed APLP1 induced cell migration phenotype (Supplementary Figures 8a-8d), further indicating APLP1 induced actin-remodeling is required for the cell migration phenotype.

## DISCUSSION

In this study we provide evidence demonstrating a direct role of APLP1 in promoting cell migration *in vivo*. This function of APLP1 depends on JNK signaling, as gain of JNK signaling boosts whereas loss of JNK signaling blocks APLP1-induced cell migration. Mechanistically, APLP1 expression induces JNK phosphorylation and subsequent activation of JNK signaling, which leads to transcriptional up-regulation of MMP1 and actin polymerization (Figure 4g). Thus, our study indicates a possible role of APLP1 in cell migration in mammals. We previously found expression of APLP1 induced caspase activation and FoxO mediated cell death in *Drosophila* [62], thus we checked if inhibiting of caspase could suppress APLP1 induced cell migration phenotype. Expression of baculovirus *p35*, which prevents cell death in *Drosophil*a [63], failed to rescue APLP1 induced cell migration phenotype (Supplementary Figures 9d) and along the A/P boundary we observed a broadened GFP labelled cells (Supplementary Figures 9d). P53 is known to interact with JNK signaling [64–67], we found that expression of a dominant negative form of P53 had no evident suppression of APLP1 induced cell migration phenotype (Supplementary Figures 9c), indicating APLP1 induced JNK-mediated cell migration independent of P53 or caspase activation. Loss of cell polarity genes, such as *scrib* and *lgl*, not only induces JNK-dependent cell migration in wing disc epithelia, but also shows oncogenic cooperation with activated Ras in eye discs to promote tumor growth and invasion [40], thus we checked if APLP1 cooperated with oncogenetic Ras in promoting tumor progression. Yet, expression of APLP1 had no significant cooperating effect with Ras in the tumor progression (Supplementary Figures 10a-10c'), indicating APLP1 induced cell migration may be through a different pathway.

**Figure 5:**
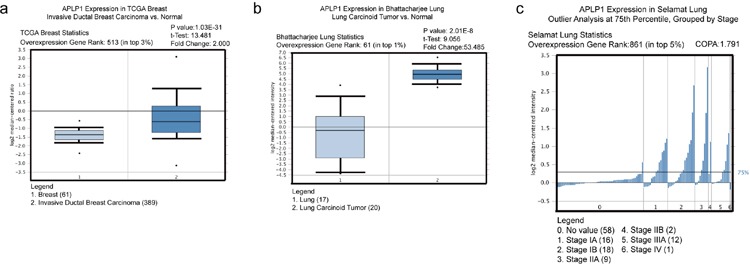
APLP1 is up-regulated in certain types of human cancer, and its higher expression may be correlated with more advanced stages in lung cancer The following data analysis all came from Oncomine database: (**a**) TCGA Breast cancer data showed that APLP1 expression was significantly up-regulated in invasive ductal breast carcinoma compared to the normal control. Fold change=2; P value=1.03E-31; (**b**) In Bhattacharjee Lung study, APLP1 expression was up-regulated significantly in lung carcinoid tumor compared to the normal control. Fold change=53.5; P value=2.01E-8; **(c**) In Selamat Lung study, outlier analysis at 75^th^ percentile was performed for APLP1 expression. APLP1 expression increased markedly from stages IA to IB and IIA above 75^th^ percentile. Sample size for stages IIB and IV was too small to be considered. COPA=1.791.

Consistent with our findings, APLP1 has been found to be significantly up-regulated in several types of human cancers including invasive ductal breast carcinoma (https://tcga-data.nci.nih.gov/docs/publications/tcga/) and lung carcinoid tumor [68] (Figure 5a and 5b). In addition, based on the outlier analysis from the Oncomine database (https://www.oncomine.org/), many more cancers, including Esophageal cancer, Ovarian cancer, Sarcoma, and Kidney cancer, have a portion of samples showing significantly higher expression of APLP1 in multiple studies, suggesting APLP1 may play an important role in some subtypes of cancers during tumorigenesis. Finally, higher APLP1 expression was observed in more advanced stages in lung cancer [69] (Figure 5c), implying a role of APLP1 in tumor metastasis.

## MATERIALS AND METHODS

### Fly genetics

All stocks were raised on standard *Drosophila* media and crosses were performed at 25°C unless otherwise indicated. *UAS*-APLP1, *UAS*-APPL^sd^ were kindly provided by Dr. Merdes [22]; *puc*^H246^, *UAS-mmp1*-IR, *UAS-mmp2*-IR, *UAS*-TIMP1, *UAS*-Ras^V12^, *UAS*-*Rho1*-IR, *UAS*-*p35*, UAs-*P53*^DN^ were obtained from Bloomington Stock Center; *sd*-Gal4, *UAS*-Bsk^DN^, *UAS*-Puc, *UAS*-Egr, *UAS*-Hep [70]; *ptc*-Gal4, *en*-Gal4, *puc*^E69 34^, *UAS*-LacZ [71] were previously described.

### Statistical analysis

For statistical analysis of cell migration, 20-30 3^rd^ instar larvae were dissected for each genotype as described [53]. Discs were separated to different categories based on the number of cells migrated to the posterior compartments.

### Immunohistochemistry

Antibody staining of the imaginal discs was performed as previously described [40]. The following antibodies were used: mouse anti-β-gal (1:400, Developmental Studies Hybridoma Bank), mouse anti-MMP1 (1:100, Developmental Studies Hybridoma Bank), rabbit anti-phospho-JNK (1:200, Calbiochem), phalloidin (1:400, Sigma). Secondary antibodies were anti-rabbit-Alexa (1:1000, Cell Signaling and Technology) and anti-mouse-Cy3 (1:1000, Jackson ImmunoResearch).

For X-gal staining, 3^rd^ instar larvae wing discs were dissected in PBST and stained for β galactosidase activity as described [72].

## SUPPLEMENTARY FIGURES



## References

[1] Wasco W, Bupp K, Magendantz M, Gusella JF, Tanzi RE, Solomon F (1992). Identification of a mouse brain cDNA that encodes a protein related to the Alzheimer disease-associated amyloid beta protein precursor. Proc Natl Acad Sci U S A.

[2] Wasco W, Gurubhagavatula S, Paradis MD, Romano DM, Sisodia SS, Hyman BT, Neve RL, Tanzi RE (1993). Isolation and characterization of APLP2 encoding a homologue of the Alzheimer's associated amyloid beta protein precursor. Nat Genet.

[3] Sprecher CA, Grant FJ, Grimm G, O'Hara PJ, Norris F, Norris K, Foster DC (1993). Molecular cloning of the cDNA for a human amyloid precursor protein homolog: evidence for a multigene family. Biochemistry.

[4] Bayer TA, Cappai R, Masters CL, Beyreuther K, Multhaup G (1999). It all sticks together--the APP-related family of proteins and Alzheimer's disease. Mol Psychiatry.

[5] Poeck B, Strauss R, Kretzschmar D (2012). Analysis of amyloid precursor protein function in Drosophila melanogaster. Exp Brain Res.

[6] Reinhard C, Hebert SS, De Strooper B (2005). The amyloid-beta precursor protein: integrating structure with biological function. Embo j.

[7] Aydin D, Weyer SW, Muller UC (2012). Functions of the APP gene family in the nervous system: insights from mouse models. Exp Brain Res.

[8] Ma QH, Futagawa T, Yang WL, Jiang XD, Zeng L, Takeda Y, Xu RX, Bagnard D, Schachner M, Furley AJ, Karagogeos D, Watanabe K, Dawe GS (2008). A TAG1-APP signalling pathway through Fe65 negatively modulates neurogenesis. Nat Cell Biol.

[9] Small DH, Nurcombe V, Reed G, Clarris H, Moir R, Beyreuther K, Masters CL (1994). A heparin-binding domain in the amyloid protein precursor of Alzheimer's disease is involved in the regulation of neurite outgrowth. J Neurosci.

[10] Perez RG, Zheng H, Van der Ploeg LH, Koo EH (1997). The beta-amyloid precursor protein of Alzheimer's disease enhances neuron viability and modulates neuronal polarity. J Neurosci.

[11] Leyssen M, Ayaz D, Hebert SS, Reeve S, De Strooper B, Hassan BA (2005). Amyloid precursor protein promotes post-developmental neurite arborization in the Drosophila brain. EMBO J.

[12] Young-Pearse TL, Chen AC, Chang R, Marquez C, Selkoe DJ (2008). Secreted APP regulates the function of full-length APP in neurite outgrowth through interaction with integrin beta1. Neural Dev.

[13] Nikolaev A, McLaughlin T, O'Leary DD, Tessier-Lavigne M (2009). APP binds DR6 to trigger axon pruning and neuron death via distinct caspases. Nature.

[14] Priller C, Bauer T, Mitteregger G, Krebs B, Kretzschmar HA, Herms J (2006). Synapse formation and function is modulated by the amyloid precursor protein. J Neurosci.

[15] Wang Z, Wang B, Yang L, Guo Q, Aithmitti N, Songyang Z, Zheng H (2009). Presynaptic and postsynaptic interaction of the amyloid precursor protein promotes peripheral and central synaptogenesis. J Neurosci.

[16] Weyer SW, Klevanski M, Delekate A, Voikar V, Aydin D, Hick M, Filippov M, Drost N, Schaller KL, Saar M, Vogt MA, Gass P, Samanta A (2011). APP and APLP2 are essential at PNS and CNS synapses for transmission, spatial learning and LTP. Embo j.

[17] Sisodia SS (2002). Biomedicine. A cargo receptor mystery APParently solved?. Science.

[18] Kamal A, Almenar-Queralt A, LeBlanc JF, Roberts EA, Goldstein LS (2001). Kinesin-mediated axonal transport of a membrane compartment containing beta-secretase and presenilin-1 requires APP. Nature.

[19] Kamal A, Stokin GB, Yang Z, Xia CH, Goldstein LS (2000). Axonal transport of amyloid precursor protein is mediated by direct binding to the kinesin light chain subunit of kinesin-I. Neuron.

[20] Gunawardena S, Goldstein LS (2001). Disruption of axonal transport and neuronal viability by amyloid precursor protein mutations in Drosophila. Neuron.

[21] Wang X, Wang Z, Chen Y, Huang X, Hu Y, Zhang R, Ho MS, Xue L (2014). FoxO mediates APP-induced AICD-dependent cell death. Cell Death Dis.

[22] Merdes G, Soba P, Loewer A, Bilic MV, Beyreuther K, Paro R (2004). Interference of human and Drosophila APP and APP-like proteins with PNS development in Drosophila. Embo j.

[23] Herms J, Anliker B, Heber S, Ring S, Fuhrmann M, Kretzschmar H, Sisodia S, Muller U (2004). Cortical dysplasia resembling human type 2 lissencephaly in mice lacking all three APP family members. Embo j.

[24] Bergmans BA, Shariati SA, Habets RL, Verstreken P, Schoonjans L, Muller U, Dotti CG, De Strooper B (2010). Neurons generated from APP/APLP1/APLP2 triple knockout embryonic stem cells behave normally in vitro and in vivo: lack of evidence for a cell autonomous role of the amyloid precursor protein in neuronal differentiation. Stem Cells.

[25] Shariati SA, Lau P, Hassan BA, Muller U, Dotti CG, De Strooper B, Gartner A (2013). APLP2 regulates neuronal stem cell differentiation during cortical development. J Cell Sci.

[26] Young-Pearse TL, Bai J, Chang R, Zheng JB, LoTurco JJ, Selkoe DJ (2007). A critical function for beta-amyloid precursor protein in neuronal migration revealed by in utero RNA interference. J Neurosci.

[27] Rice HC, Townsend M, Bai J, Suth S, Cavanaugh W, Selkoe DJ, Young-Pearse TL (2012). Pancortins interact with amyloid precursor protein and modulate cortical cell migration. Development.

[28] Li XF, Thinakaran G, Sisodia SS, Yu FS (1999). Amyloid precursor-like protein 2 promotes cell migration toward fibronectin and collagen IV. J Biol Chem.

[29] Kaden D, Voigt P, Munter LM, Bobowski KD, Schaefer M, Multhaup G (2009). Subcellular localization and dimerization of APLP1 are strikingly different from APP and APLP2. J Cell Sci.

[30] Adlerz L, Beckman M, Holback S, Tehranian R, Cortes Toro V, Iverfeldt K (2003). Accumulation of the amyloid precursor-like protein APLP2 and reduction of APLP1 in retinoic acid-differentiated human neuroblastoma cells upon curcumin-induced neurite retraction. Brain Res Mol Brain Res.

[31] Tang X, Milyavsky M, Goldfinger N, Rotter V (2007). Amyloid-beta precursor-like protein APLP1 is a novel p53 transcriptional target gene that augments neuroblastoma cell death upon genotoxic stress. Oncogene.

[32] Arvidsson Y, Andersson E, Bergstrom A, Andersson MK, Altiparmak G, Illerskog AC, Ahlman H, Lamazhapova D, Nilsson O (2008). Amyloid precursor-like protein 1 is differentially upregulated in neuroendocrine tumours of the gastrointestinal tract. Endocr Relat Cancer.

[33] Ke H, Augustine CK, Gandham VD, Jin JY, Tyler DS, Akiyama SK, Hall RP, Zhang JY (2013). CYLD inhibits melanoma growth and progression through suppression of the JNK/AP-1 and beta1-integrin signaling pathways. J Invest Dermatol.

[34] Ma X, Shao Y, Zheng H, Li M, Li W, Xue L (2013). Src42A modulates tumor invasion and cell death via Ben/dUev1a-mediated JNK activation in Drosophila. Cell Death Dis.

[35] Hong YK, Lee S, Park SH, Lee JH, Han SY, Kim ST, Kim YK, Jeon S, Koo BS, Cho KS (2012). Inhibition of JNK/dFOXO pathway and caspases rescues neurological impairments in Drosophila Alzheimer's disease model. Biochem Biophys Res Commun.

[36] Tare M, Modi RM, Nainaparampil JJ, Puli OR, Bedi S, Fernandez-Funez P, Kango-Singh M, Singh A (2011). Activation of JNK signaling mediates amyloid-ss-dependent cell death. PLoS One.

[37] Vidal M, Larson DE, Cagan RL (2006). Csk-deficient boundary cells are eliminated from normal Drosophila epithelia by exclusion, migration, and apoptosis. Dev Cell.

[38] Das TK, Sangodkar J, Negre N, Narla G, Cagan RL (2013). Sin3a acts through a multi-gene module to regulate invasion in Drosophila and human tumors. Oncogene.

[39] Cordero JB, Macagno JP, Stefanatos RK, Strathdee KE, Cagan RL, Vidal M (2010). Oncogenic Ras diverts a host TNF tumor suppressor activity into tumor promoter. Dev Cell.

[40] Igaki T, Pagliarini RA, Xu T (2006). Loss of cell polarity drives tumor growth and invasion through JNK activation in Drosophila. Curr Biol.

[41] Igaki T, Kanda H, Yamamoto-Goto Y, Kanuka H, Kuranaga E, Aigaki T, Miura M (2002). Eiger, a TNF superfamily ligand that triggers the Drosophila JNK pathway. Embo j.

[42] Moreno E, Yan M, Basler K (2002). Evolution of TNF signaling mechanisms: JNK-dependent apoptosis triggered by Eiger, the Drosophila homolog of the TNF superfamily. Curr Biol.

[43] Glise B, Bourbon H, Noselli S (1995). hemipterous encodes a novel Drosophila MAP kinase kinase, required for epithelial cell sheet movement. Cell.

[44] Martin-Blanco E, Gampel A, Ring J, Virdee K, Kirov N, Tolkovsky AM, Martinez-Arias A (1998). puckered encodes a phosphatase that mediates a feedback loop regulating JNK activity during dorsal closure in Drosophila. Genes Dev.

[45] Xue L, Igaki T, Kuranaga E, Kanda H, Miura M, Xu T (2007). Tumor suppressor CYLD regulates JNK-induced cell death in Drosophila. Dev Cell.

[46] Sluss HK, Han Z, Barrett T, Goberdhan DC, Wilson C, Davis RJ, Ip YT (1996). A JNK signal transduction pathway that mediates morphogenesis and an immune response in Drosophila. Genes Dev.

[47] Rosen DR, Martin-Morris L, Luo LQ, White K (1989). A Drosophila gene encoding a protein resembling the human beta-amyloid protein precursor. Proc Natl Acad Sci U S A.

[48] Agnes F, Suzanne M, Noselli S (1999). The Drosophila JNK pathway controls the morphogenesis of imaginal discs during metamorphosis. Development.

[49] Beaucher M, Hersperger E, Page-McCaw A, Shearn A (2007). Metastatic ability of Drosophila tumors depends on MMP activity. Dev Biol.

[50] Deryugina EI, Quigley JP (2006). Matrix metalloproteinases and tumor metastasis. Cancer Metastasis Rev.

[51] Uhlirova M, Bohmann D (2006). JNK- and Fos-regulated Mmp1 expression cooperates with Ras to induce invasive tumors in Drosophila. EMBO J.

[52] Srivastava A, Pastor-Pareja JC, Igaki T, Pagliarini R, Xu T (2007). Basement membrane remodeling is essential for Drosophila disc eversion and tumor invasion. Proc Natl Acad Sci U S A.

[53] Rudrapatna VA, Bangi E, Cagan RL (2013). Caspase signalling in the absence of apoptosis drives Jnk-dependent invasion. EMBO Rep.

[54] Rudrapatna VA, Bangi E, Cagan RL (2013). A Jnk-Rho-Actin remodeling positive feedback network directs Src-driven invasion. Oncogene.

[55] Verderame M, Alcorta D, Egnor M, Smith K, Pollack R (1980). Cytoskeletal F-actin patterns quantitated with fluorescein isothiocyanate-phalloidin in normal and transformed cells. Proc Natl Acad Sci U S A.

[56] Benlali A, Draskovic I, Hazelett DJ, Treisman JE (2000). act up controls actin polymerization to alter cell shape and restrict Hedgehog signaling in the Drosophila eye disc. Cell.

[57] Contasta I, Berghella AM, Pellegrini P, Del Beato T, Casciani CA, Adorno D (1999). Relationships between the activity of MMP1/TIMP1 enzymes and the TH1/TH2 cytokine network. Cancer Biother Radiopharm.

[58] Markiewicz L, Majsterek I, Przybylowska K, Dziki L, Waszczyk M, Gacek M, Kaminska A, Szaflik J, Szaflik JP (2013). Gene polymorphisms of the MMP1, MMP9, MMP12, IL-1beta and TIMP1 and the risk of primary open-angle glaucoma. Acta Ophthalmol.

[59] Ouyang QC, Hu CP, Liang QH (2003). [Gene expression of MMP1 and TIMP1 in lung cancer detected with a cDNA microarray technique]. [Article in Chinese]. Hunan Yi Ke Da Xue Xue Bao.

[60] Magie CR, Meyer MR, Gorsuch MS, Parkhurst SM (1999). Mutations in the Rho1 small GTPase disrupt morphogenesis and segmentation during early Drosophila development. Development.

[61] Ma X, Chen Y, Zhang S, Xu W, Shao Y, Yang Y, Li W, Li M, Xue L (2016). Rho1-Wnd signaling regulates loss-of-cell polarity-induced cell invasion in Drosophila. Oncogene.

[62] Wang X, Ma Y, Zhao Y, Chen Y, Hu Y, Chen C, Shao Y, Xue L (2015). APLP1 promotes dFoxO-dependent cell death in Drosophila. Apoptosis.

[63] Hay BA, Wolff T, Rubin GM (1994). Expression of baculovirus P35 prevents cell death in Drosophila. Development.

[64] Wang J, Friedman E (2000). Downregulation of p53 by sustained JNK activation during apoptosis. Mol Carcinog.

[65] Fuchs SY, Adler V, Pincus MR, Ronai Z (1998). MEKK1/JNK signaling stabilizes and activates p53. Proc Natl Acad Sci U S A.

[66] Topisirovic I, Gutierrez GJ, Chen M, Appella E, Borden KL, Ronai ZA (2009). Control of p53 multimerization by Ubc13 is JNK-regulated. Proc Natl Acad Sci U S A.

[67] Tafolla E, Wang S, Wong B, Leong J, Kapila YL (2005). JNK1 and JNK2 oppositely regulate p53 in signaling linked to apoptosis triggered by an altered fibronectin matrix: JNK links FAK and p53. J Biol Chem.

[68] Bhattacharjee A, Richards WG, Staunton J, Li C, Monti S, Vasa P, Ladd C, Beheshti J, Bueno R, Gillette M, Loda M, Weber G, Mark EJ (2001). Classification of human lung carcinomas by mRNA expression profiling reveals distinct adenocarcinoma subclasses. Proc Natl Acad Sci U S A.

[69] Selamat SA, Chung BS, Girard L, Zhang W, Zhang Y, Campan M, Siegmund KD, Koss MN, Hagen JA, Lam WL, Lam S, Gazdar AF, Laird-Offringa IA (2012). Genome-scale analysis of DNA methylation in lung adenocarcinoma and integration with mRNA expression. Genome Res.

[70] Ma X, Huang J, Yang L, Yang Y, Li W, Xue L (2012). NOPO modulates Egr-induced JNK-independent cell death in Drosophila. Cell Res.

[71] Li WZ, Li SL, Zheng HY, Zhang SP, Xue L (2012). A broad expression profile of the GMR-GAL4 driver in Drosophila melanogaster. Genet Mol Res.

[72] Xue L, Noll M (2000). Drosophila female sexual behavior induced by sterile males showing copulation complementation. Proc Natl Acad Sci U S A.

