# Supplementary material for: Amyloid precursor like protein-1 promotes JNK-mediated cell migration in Drosophila
Source: Oncotarget. 2017 May 8;8(30):49725–34. doi: 10.18632/oncotarget.17681 (PMC5564802; doi:10.18632/oncotarget.17681)
Supplement: Supplementary file 1 [file oncotarget-08-49725-s001.pdf]

Amyloid precursor like protein-1 promotes JNK-mediated cell migration in *Drosophila*

SUPPLEMENTARY MATERIALS

SUPPLEMENTARY FIGURES

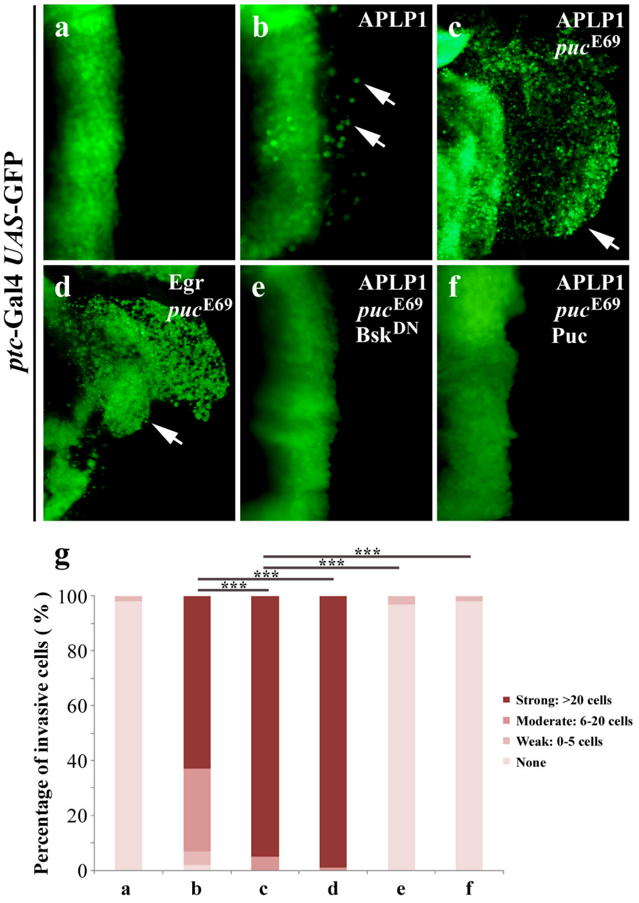

Supplementary Figure 1: Inactivation of JNK signaling suppressed APLP1-induced *puc*^{E69}-enhanced cell migration. Fluorescence micrographs of wing disc are shown. Compared with the *ptc*-Gal4 *UAS*-GFP control (**a**), APLP1-induced cell migration (**b**) was significantly enhanced in heterozygous *puc* mutants (**c**), which was fully suppressed by expression of Bsk^{DN} (**e**)or Puc (**f**). Egr-induced cell migration was also enhanced (**d**). **(g)** Quantification of migration phenotype in a-f. ***, P<0.001. The crosses were performed at 29°C. The genotypes used in the figure are as follows: *ptc*-Gal4 *UAS*-GFP/+ (**a**), *ptc*-Gal4 *UAS*-GFP/+; *UAS*-APLP1/+ (**b**), *ptc*-Gal4 *UAS*-GFP/+; *UAS*-APLP1/*puc*^{E69} (**c**), *ptc*-Gal4 *UAS*-GFP/*UAS*-Egr; *puc*^{E69}/+ (**d**), *ptc*-Gal4 *UAS*-GFP/+; *UAS*-APLP1 *UAS*-Bsk^{DN}/*puc*^{E69} (**e**), *ptc*-Gal4 *UAS*-GFP/+; *UAS*-APLP1 *UAS*-Puc/*puc*^{E69} (**f**).

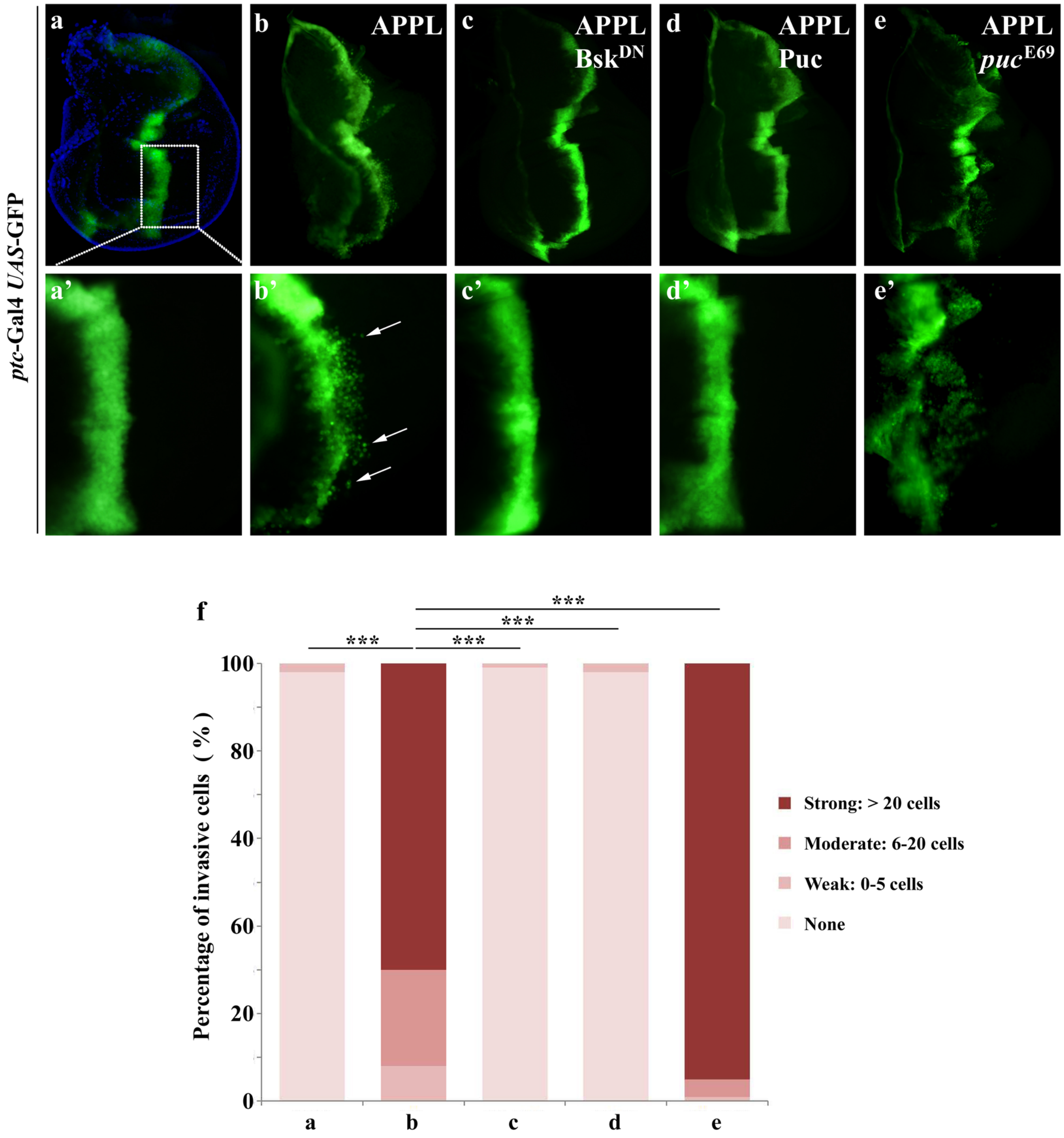

Supplementary Figure 2: APPL induced JNK-mediated cell migration. Fluorescence micrographs of wing discs are shown. Compared with the *ptc*-Gal4 *UAS*-GFP control (**a**, **a'**), APPL-induced cell migration (**b, b'**) was blocked by expression of Bsk^{DN} (**c, c'**) or Puc (**d, d'**), and enhanced in the *puc* mutant background (**e, e'**). (**f**) Quantification of migration phenotype in **a**-**e**. The crosses were performed at 29°C. ***, P <0.001. The genotypes used in the figure are as follows: *ptc*-Gal4 *UAS*-GFP/+ (**a**, **a'**), *ptc*-Gal4 *UAS*-GFP/*UAS*-$APPL^{sd}$ (**b, b'**), *ptc*-Gal4 *UAS*-GFP/*UAS*-$APPL^{sd}$; *UAS*-Bsk^{DN}/+ (**c, c'**), *ptc*-Gal4 *UAS*-GFP/*UAS*-$APPL^{sd}$; *UAS*-Puc/+ (**d, d'**), *ptc*-Gal4 *UAS*-GFP/*UAS*-$APPL^{sd}$; puc^{E69}/+ (**e, e'**).

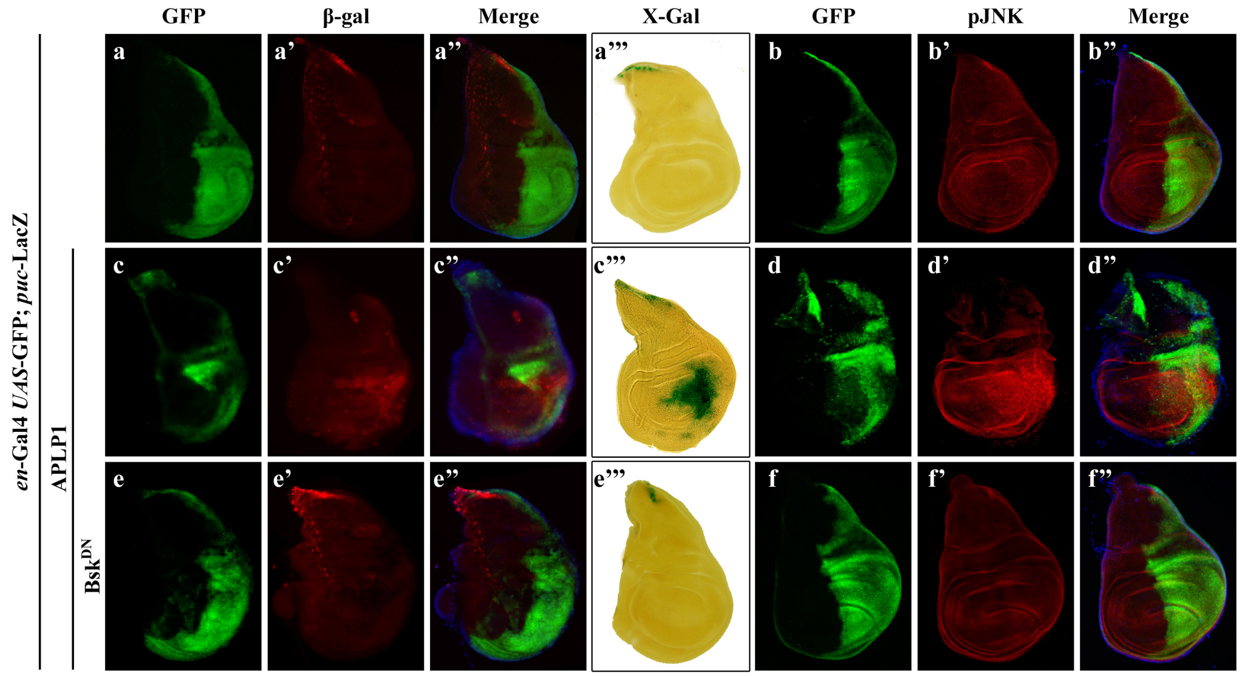

Supplementary Figure 3: APLP1 induces JNK phosphorylation and *puc* expression in the posterior compartment of wing discs. Compared with the *en*-Gal4 *UAS*-GFP control (**a-a'''**, **b-b''**), expression of APLP1 in the posterior compartment of wing discs activated *puc* expression, detected by β-gal antibody (**c-c''**) or X-gal staining (**c'''**), and JNK phosphorylation (**d-d''**), both were suppressed by expression of Bsk^{DN} (**e-e'''**, **f-f''**). The crosses were performed at 29°C. The genotypes used in the figure are as follows: *en*-Gal4 *UAS*-GFP/+; *puc*-LacZ/+ (**a-a'''**, **b-b''**), *en*-Gal4 *UAS*-GFP/+; *puc*-LacZ/*UAS*-APLP1 (**c-c'''**, **d-d''**), *en*-Gal4 *UAS*-GFP/+; *puc*-LacZ/*UAS*-APLP1 *UAS*-Bsk^{DN} (**e-e'''**, **f-f''**).

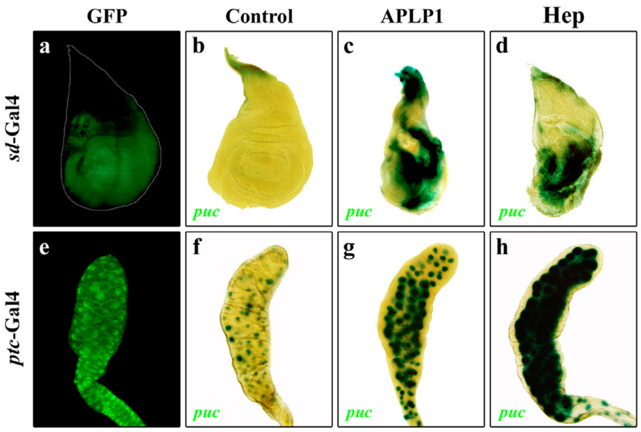

Supplementary Figure 4: APLP1 induces *puc* expression in the wing pouch and salivary gland. Fluorescence (**a**, **e**) and light micrographs (**b-d**, **f-h**) of wing disc (**a-d**) and salivary gland (**e-h**) are shown. The expression patterns of *sd*-Gal4 in the wing pouch (**a**) and *ptc*-Gal4 in the salivary gland (**e**) were shown by GFP. Compared with the control (**b**, **f**), expression of APLP1 (**c**, **g**) or Hep (**d**, **h**) induced *puc* expression in the wing pouch (**c**, **d**) and salivary gland (**g**, **h**). The crosses were performed at 25°C. The genotypes used in the figure are as follows: *sd*-Gal4/+; *UAS*-GFP/+ (**a**), *sd*-Gal4/+; *puc*-LacZ/+ (**b**), *sd*-Gal4/+; *puc*-LacZ/*UAS*-APLP1 (**c**), *sd*-Gal4/+; *UAS*-Hep/+; *puc*-LacZ/+ (**d**), *ptc*-Gal4 *UAS*-GFP/+ (**e**), *ptc*-Gal4 *UAS*-GFP/+; *puc*-LacZ/+ (**f**), *ptc*-Gal4 *UAS*-GFP/+; *puc*-LacZ/*UAS*-APLP1 (**g**), *ptc*-Gal4 *UAS*-GFP/*UAS*-Hep; *puc*-LacZ/+ (**h**).

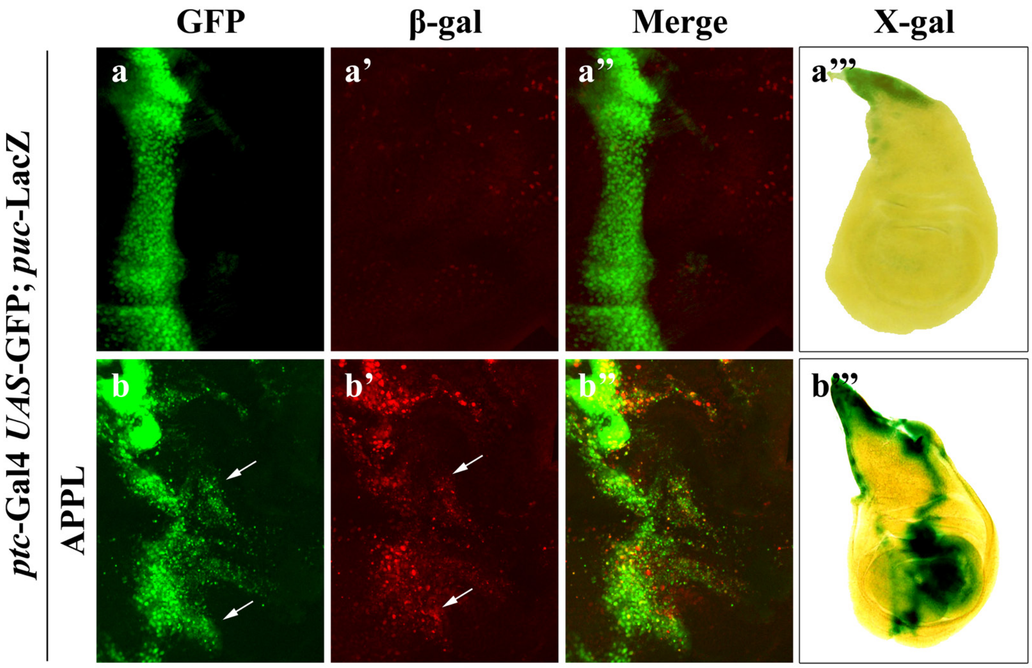

Supplementary Figure 5: APPL induces *puc* expression in the wing disc. Fluorescence (**a-a"**, **b-b"**) and light micrographs (**a'"**, **b'"**) of wing disc are shown. Compared with the control (**a-a'"**), expression of APPL (**b-b'"**) induced *puc* expression along the A/P boundary in the wing disc. The crosses were performed at 29°C. The genotypes used in the figure are as follows: *ptc*-Gal4 *UAS*-GFP/+ ; *puc*-LacZ/+ (**a-a'"**), *ptc*-Gal4 *UAS*-GFP/*UAS*-APPL^{sd} ; *puc*-LacZ/+ (**b-b'"**).

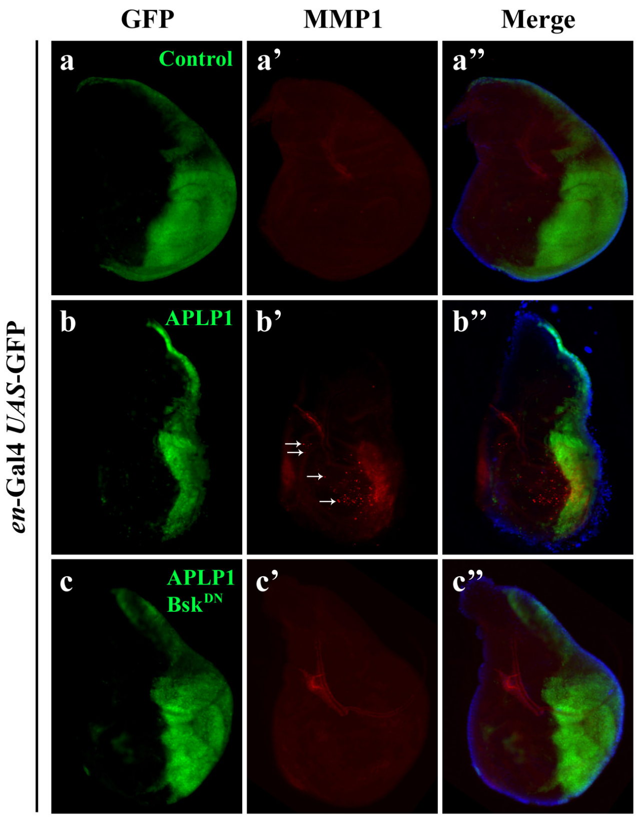

Supplementary Figure 6: APLP1 induces JNK-dependent MMP1 expression in the posterior compartment of wing discs. Fluorescence micrographs of wing disc are shown. Compared with the *en*-Gal4 *UAS*-GFP control (**a-a"**), expression of APLP1 in the posterior compartment of wing disc driven by *en*-Gal4 activated MMP1 expression (**b-b"**), which was blocked by inactivation of JNK signaling (**c-c"**). Scattered MMP1 expression was also detected in the anterior compartment of the discs (arrow). The crosses were performed at 25°C. The genotypes used in the figure are as follows: *en*-Gal4 *UAS*-GFP/+; *puc*-LacZ/+ (**a-a"**), *en*-Gal4 *UAS*-GFP/+; *puc*-LacZ/*UAS*-APLP1 (**b-b"**), *en*-Gal4 *UAS*-GFP/+; *puc*-LacZ/*UAS*-APLP1 *UAS*-Bsk^{DN} (**c-c"**).

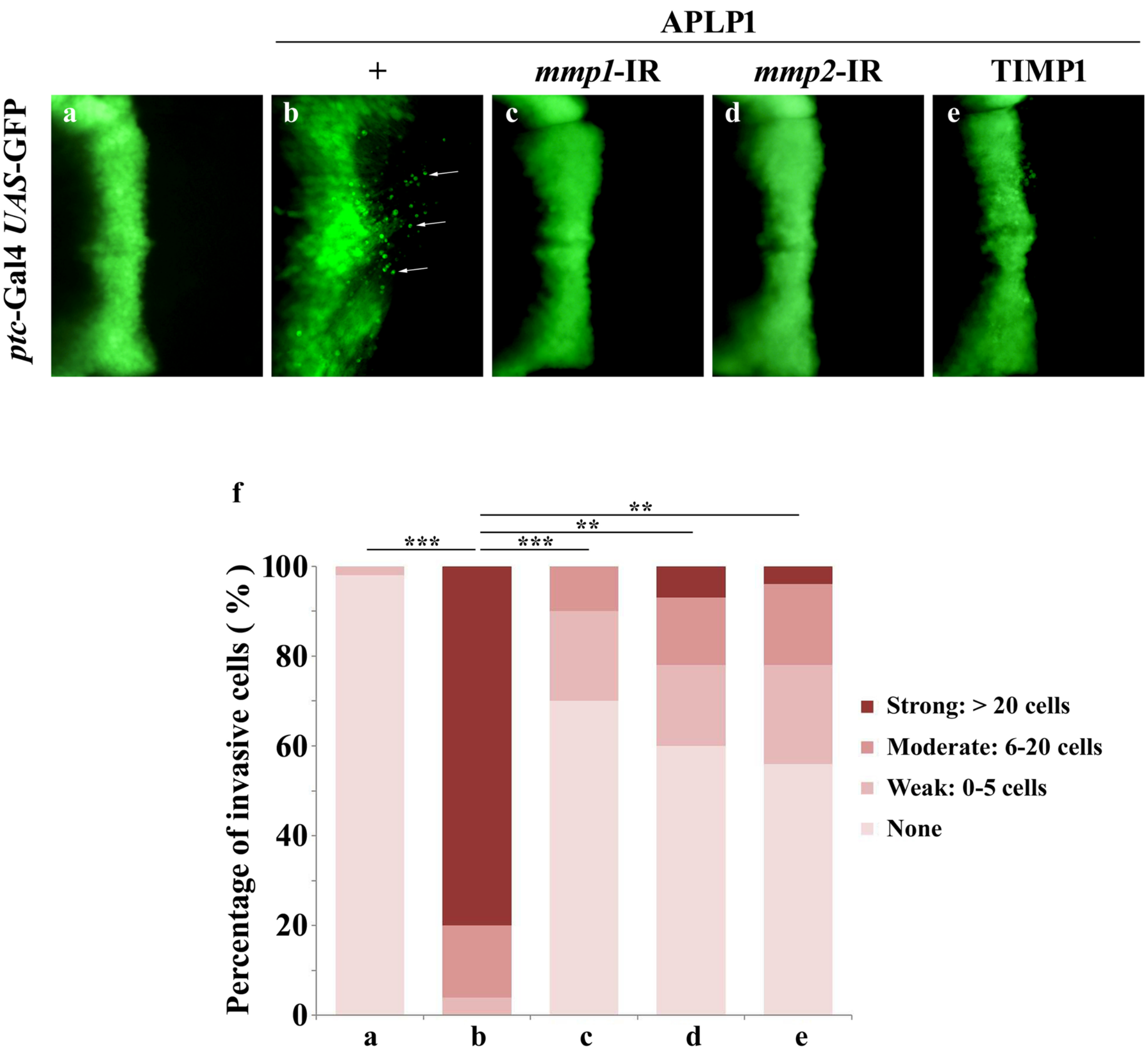

Supplementary Figure 7: Loss the MMP1 compromised APLP1 induced cell migration. Fluorescence micrographs of wing discs are shown. Compared with the *ptc*-Gal4 *UAS*-GFP control (**a**), APLP1-induced cell migration (**b**) was compromised by expression of *mmp1*-IR (**c**), *mmp2*-IR (**d**) or TIMP1 (**e**). (**f**) Quantification of migration phenotype in **a**-**e**. The crosses were performed at 29°C. ***, P <0.001, **, P <0.01. The genotypes used in the figure are as follows: *ptc*-Gal4 *UAS*-GFP/+ (**a**), *ptc*-Gal4 *UAS*-GFP/+; *UAS*-APLP1/+ (**b**), *ptc*-Gal4 *UAS*-GFP/+; *UAS*-APLP1/*UAS-mmp1-IR* (**c**), *ptc*-Gal4 *UAS*-GFP/+; *UAS*-APLP1/*UAS-mmp2-IR* (**d**), *ptc*-Gal4 *UAS*-GFP/+; *UAS*-APLP1/*UAS*-TIMP1 (**e**).

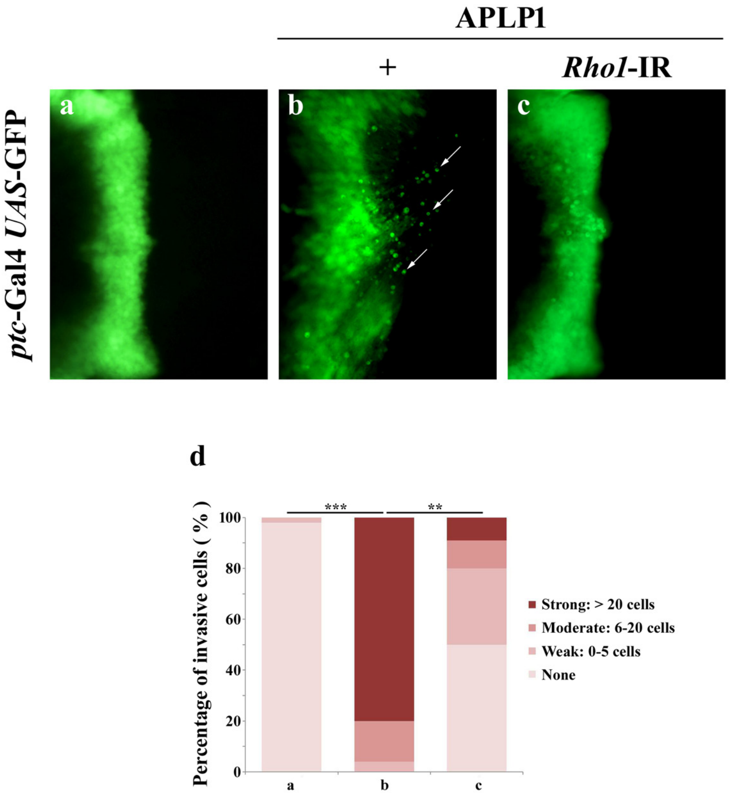

Supplementary Figure 8: Loss the Rho1 compromised APLP1 induced cell migration. Fluorescence micrographs of wing discs are shown. Compared with the *ptc*-Gal4 *UAS*-GFP control (**a**), APLP1-induced cell migration (**b**) was compromised by expression of *Rho1*-IR (**c**). (**d**) Quantification of migration phenotype in **a**-**c**. The crosses were performed at 29°C. ***, P <0.001, **, P <0.01. The genotypes used in the figure are as follows: *ptc*-Gal4 *UAS*-GFP/+ (**a**), *ptc*-Gal4 *UAS*-GFP/+; *UAS*-APLP1/+ (**b**), *ptc*-Gal4 *UAS*-GFP/+; *UAS*-APLP1/*UAS*-*Rho1*-*IR* (**c**).

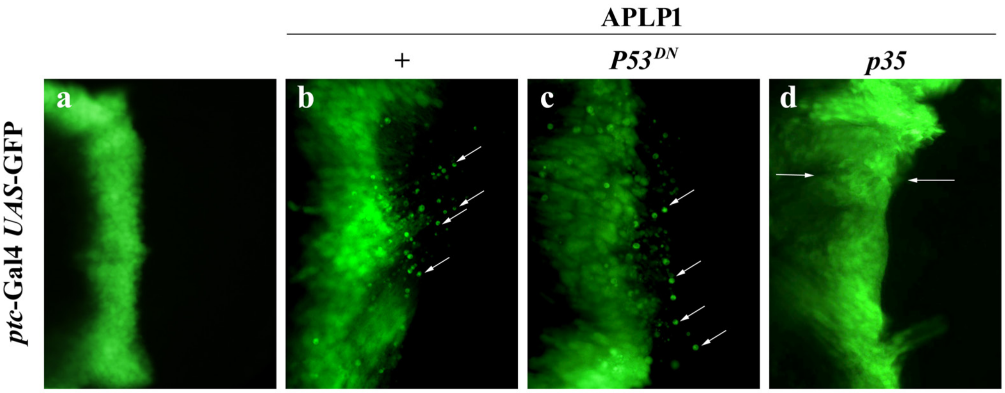

Supplementary Figure 9: APLP1 induced cell migration is independent of P53 and caspase. Fluorescence micrographs of wing discs are shown. Compared with the *ptc*-Gal4 *UAS*-GFP control (**a**), APLP1-induced cell migration (**b**) was not blocked by expression of $P53^{DN}$ (**c**) or *p35* (**d**). The crosses were performed at 29°C. The genotypes used in the figure are as follows: *ptc*-Gal4 *UAS*-GFP/+ (**a**), *ptc*-Gal4 *UAS*-GFP/+; *UAS*-APLP1/+ (**b**), *ptc*-Gal4 *UAS*-GFP/+; *UAS*-APLP1/*UAS*-$P53^{DN}$ (**c**), *ptc*-Gal4 *UAS*-GFP/+; *UAS*-APLP1/*UAS*-*p35* (**d**).

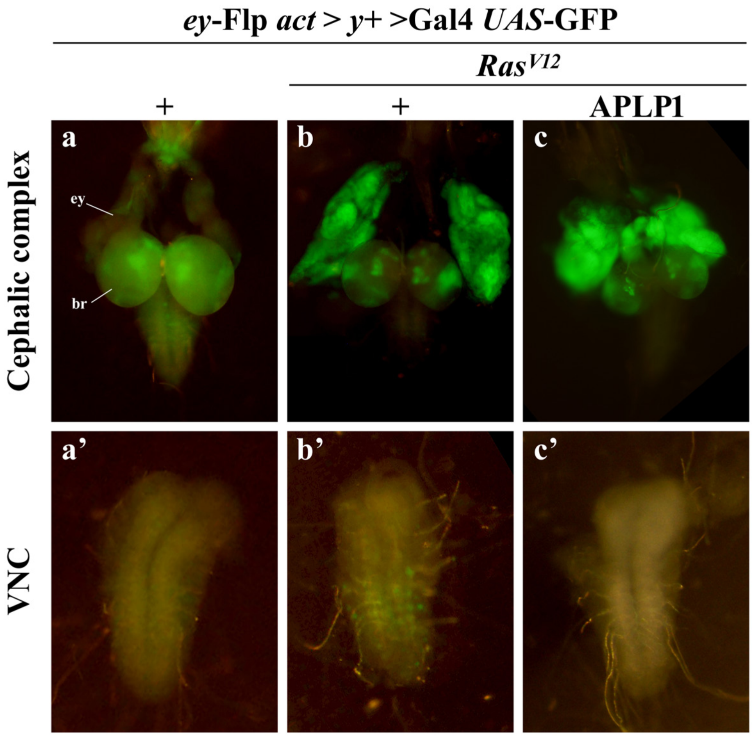

Supplementary Figure 10: APLP1 showed no significant cooperating effect with Ras in the tumor progression. **a-c'**, Clones of cells marked with GFP in the eye-antennal discs of third-instar larvae are shown. Upper panels show the cephalic complex (CC) (**a-c**), which consists of eye-antennal discs (ey), brain (br) and ventral nerve cord (VNC). Lower panels show the dissected VNC (**a'-c'**). Compared to wild-type clones (**a**), Ras^{V12} overexpressing clones overgrow moderately (**b**). Co-expression of APLP1 and Ras^{V12} failed to invade the VNC (**a'-c'**). The crosses were performed at 25°C. The genotypes used in the figure are as follows: *ey*-Flp *act*>*y*+>Gal4 *UAS*-GFP/+ (**a**), *ey*-Flp *act*>*y*+>Gal4 *UAS*-GFP/*UAS-Ras^{V12}* (**b**), *ey*-Flp *act*>*y*+>Gal4 *UAS*-GFP/*UAS-Ras^{V12}*; *UAS*-APLP1/+ (**c**).
